# *Atg5* deficiency in macrophages protects against kidney fibrosis via the CCR6-CCL20 axis

**DOI:** 10.1186/s12964-024-01600-2

**Published:** 2024-04-09

**Authors:** Yufeng Zhu, Jiexing Tan, Yuanzhan Wang, Yuhong Gong, Xiaoyong Zhang, Ziguo Yuan, Xinyu Lu, Huifang Tang, Zhiming Zhang, Xiaotao Jiang, Wei Zhu, Li Gong

**Affiliations:** 1 Experimental Animal Center, Nanfang Hospital, Southern Medical University, No. 1838, North Guangzhou Avenue, Baiyun District, Guangzhou, 510515 China; 2 State Key Laboratory of Organ Failure Research, Guangdong Provincial Key Laboratory of Viral Hepatitis Research, Department of Infectious Diseases, Nanfang Hospital, Southern Medical University, Guangzhou, Guangdong China; 3 College of Veterinary Medicine, South China Agricultural University, Guangzhou, China; 4 Guangdong Provincial Key Laboratory of Zoonosis Prevention and Control, College of Veterinary Medicine, South China Agricultural University, Guangzhou, China; 5 Department of Infectious Diseases, Nanfang Hospital, Southern Medical University, Guangzhou, China; 6 Department of Pharmacology, School of Basic Medical Sciences, Zhejiang University, Hangzhou, China; 7 Department of Immunology, School of Basic Medical Sciences, Southern Medical University, Guangzhou, China; 8 Guangdong Provincial Key Laboratory of Proteomics, Guangzhou, China

**Keywords:** Autophagy-related 5, Autophagy, Acute kidney injury, Macrophage, Renal fibrosis

## Abstract

**Background:**

Autophagy is a lysosome-dependent degradation pathway that regulates macrophage activation, differentiation, and polarization. Autophagy related 5 (Atg5) is a key protein involved in phagocytic membrane elongation in autophagic vesicles that forms a complex with Atg12 and Atg16L1. Alterations in *Atg5* are related to both acute and chronic kidney diseases in experimental models. However, the role of macrophage-expressed *Atg5* in acute kidney injury remains unclear.

**Methods:**

Using a myeloid cell-specific *Atg5* knockout (*MΦ atg5*^−/−^) mouse, we established renal ischemia/reperfusion and unilateral ureteral obstruction models to evaluate the role of macrophage *Atg5* in renal macrophage migration and fibrosis.

**Results:**

Based on changes in the serum urea nitrogen and creatinine levels, *Atg5* deletion had a minimal effect on renal function in the early stages after mild injury; however, M*Φ atg5*^−/−^ mice had reduced renal fibrosis and reduced macrophage recruitment after 4 weeks of ischemia/reperfusion injury and 2 weeks of unilateral ureteral obstruction injury. *Atg5* deficiency impaired the CCL20-CCR6 axis after severe ischemic kidneys. Chemotactic responses of bone marrow-derived monocytes (BMDMs) from MΦ *atg5*^−/−^ mice to CCL20 were significantly attenuated compared with those of wild-type BMDMs, and this might be caused by the inhibition of PI3K, AKT, and ERK1/2 activation.

**Conclusions:**

Our data indicate that *Atg5* deficiency decreased macrophage migration by impairing the CCL20-CCR6 axis and inhibited M2 polarization, thereby improving kidney fibrosis.

**Supplementary Information:**

The online version contains supplementary material available at 10.1186/s12964-024-01600-2.

## Background

Acute kidney injury (AKI) is a global public health issue that is characterized by kidney function loss, leading to high morbidity and mortality rates [1]. Currently, no therapeutic approaches can prevent injury, improve survival, or accelerate recovery upon AKI onset. Additionally, insufficient AKI recovery leads to chronic kidney injury [2–5]. After the acute phase, persistent maladaptive repair promotes inflammation and fibrosis in the chronic phase. Furthermore, the activation of effector cells by inflammatory cells is generally considered the trigger for fibrosis. Macrophages play a critical role in the translation of injury to abnormal repair in renal fibrosis, which is an important determinant of AKI outcomes [6–11].

Autophagy is a highly conserved lysosome-dependent mechanism. Senescent organelles or abnormal proteins are transported by cells to lysosomes, where they are degraded by mediating multiple autophagy-related genes and complex cellular signaling pathways [12, 13]. Atg5 (autophagy related 5) is involved in phagocytic membrane elongation in autophagic vesicles and forms a complex with Atg12 and Atg16L1 [14]. Many evidence suggests that Atg5 in kidney tubules is involved in kidney repair during transition from AKI to chronic kidney disease (CKD), Tubular specific *Atg5* knockout (KO) mice exhibit aggravated IRI or cisplatin induced renal injury and cell apoptosis. *Atg5*-deficient cells accumulated deformed mitochondria, increased ROS production, DNA damage, proximal tubule cell apoptosis, and loss of renal function. In addition, Baisantry et al. demonstrated that *Atg5* deficient renal tubular cells can attenuate kidney injury after ischemic AKI, reduce inflammation and interstitial fibrosis. Therefore, it remains controversial whether Atg5 promotes or inhibits fibrosis [15–18].

Rapamycin is an activator of autophagy by inhibiting the mechanism target of rapamycin complex 1 (mTORC1) [19]. Zhang et al. found that rapamycin inhibited M1 macrophage polarization and renal fibrosis by activating macrophage autophagy [20]. Treatment of macrophages with an autophagy agonist reportedly ameliorated lipopolysaccharide (LPS)-induced AKI, consequently inhibiting the secretion of inflammatory factors, whereas the inhibition of macrophage autophagy promoted the release of inflammatory factors [21]. These results indicate that intervening in macrophage autophagy can affect its polarization, chronic inflammation and ultimately fibrosis. However, the role of Atg5 in macrophage related to AKI or kidney fibrosis are unclear.

Therefore, this study aimed to investigate the function of *Atg5* using mouse models of ischemia/reperfusion (I/R) injury and unilateral ureteral obstruction (UUO). We further explored whether deletion of myeloid *Atg5* impacts recovery from renal ischemic injury and promotes subsequent renal fibrosis.

## Methods

### Animals

All animal experiments complied with a protocol approved by the Institutional Animal Care and Use Committee of Nanfang Hospital Southern Medical University. *Atg5*^*flox/flox*^ and *LysM-Cre* mice were obtained from a C57BL/6 background. *Atg5*^*flox/flox*^ and *LysM-Cre* mice were purchased from RIKEN BioResource Research Center (RBRC02975) and Jackson Laboratory (004781), respectively. *Atg5*^*flox/flox*^ mice were crossed with *LysM-Cre*^*+*^ mice to obtain *LysM-Cre*^*+*^*Atg5*^*flox/flox*^ macrophage-conditional *Atg5* knockout (MΦ *atg5*^*−/−*^) and *LysM-Cre*^*–*^*Atg5*^*flox/flox*^ (WT) littermate mice. All mice were genotyped using PCR before and after all experiments. Genotyping of genomic DNA from the tail of a 4-week-old mouse was performed using PCR amplification with primers *Atg5*^*flox/flox*^ (5’-GAATATGAAGGCACACCCCTGAAATG-3’, 5’-ACAACGTCGAGCACAGCTGCGCAAGG-3’, and 5’-GTACTGCATAATGGTTTAACTCT TGC-3’) and *LysM-Cre* (5’-CCCAGAAATGCCAGATTACG-3’, 5’-CTTGGGCTGCCAGA ATTTCTC-3’, and 5’-TTACAGTCGGCCAGGCTGAC-3’). *Atg5*^*flox/flox*^ amplified a 700-bp fragment, while *LysM-Cre*^*+/−*^ amplification of a 350-bp fragment and a 700-bp fragment. The mice were bred and maintained under pathogen-free conditions at an experimental animal center (Nanfang Hospital) under a natural light cycle with free access to water and food. Specifically, 8–10-week-old male mice were used in this study and subjected to surgical procedures.

### Establishment of I/R and UUO mouse models

First, the mice were intraperitoneally anesthetized with barbital sodium (60 mg/kg) for the kidney I/R models. During surgery, a homeothermic blanket control unit with a rectal probe (Shanghai Yuyan Scientific Instrument Co., Shanghai, China, Y585650809) was used to maintain body temperature at 36.5 °C. The kidneys were subjected to bilateral flank incisions, and both pedicles were clamped for 26 min. After the clamp was released, color changes in the kidneys were noted, which suggested adequate ischemia and reperfusion. Animals were sacrificed at different times (1 day, 4 days, and 7 days). Therefore, to induce unilateral renal I/R, the left renal pedicle was clamped for 31 min. Subsequently, the clamp was released for reperfusion at different durations (14 and 28 days). In contrast, sham control mice underwent the same procedure without a clamped renal pedicle. In a part of the unilateral ischemia AKI experiments, left renal reperfusion was performed for 13 days, after which the right kidney was removed, and blood samples were collected 1 day later to determine blood urea nitrogen (BUN) and serum creatinine levels to evaluate left kidney function. For the UUO models, the left abdominal cavity of the mouse was opened, the left ureter was exposed and separated, and the middle ureter was ligated with a silk thread. Finally, the mice were sacrificed 14 days after UUO, and the contralateral and obstructed kidneys were harvested for further analysis.

### Flow cytometry

Kidney cell suspensions were prepared from the mice subjected to I/R, UUO, or sham surgery. The kidneys were perfused with phosphate-buffered saline (PBS), minced into fragments, filtered through a 40-µm filter, and digested in Gey’s balanced salt solution containing 0.7 mg/mL Liberase (Roche, 5,401,127,001) for 30 min at 37 °C to obtain a single-cell suspension. Next, red blood cells were removed using erythrocyte lysis buffer (QIAGEN, 79,217). The cells were blocked in 2.5 mg/mL Fc blocking solution and then stained with the fluorescent antibodies PerCP/Cyanine5.5 anti-mouse CD45.2 (109,827), PE anti-mouse F4/80 (123,110), FITC anti-mouse CD11c (117,306), PE/Cyanine7 anti-mouse CD206 (141,720), and APC/Cy7 anti-mouse CD11b (557,657). These antibodies were purchased from BioLegend, except for APC/Cy7 anti-mouse CD11b, which was purchased from BD Biosciences. Macrophages were identified as CD45^+^ CD11c^−^ CD11b^+^ F4/80^+^ cells. PE/Cyanine7 anti-mouse CD206 antibody was used to identify M2 macrophage polarization. Flow cytometry data were acquired on the BD Canto II system (BD Bioscience, Franklin Lakes, NJ) and analyzed using FCS Express 10.0 software.

### Renal function, histology, immunohistochemistry, and quantitative image analysis

Blood samples were collected at the indicated time points to examine renal function. Whole blood was allowed to settle at room temperature (∽ 25 °C) for 30 min. After coagulation, the blood samples were centrifuged at 1,000 × *g* for 10 min at 4 °C, and the supernatant was collected as the serum sample. BUN and serum creatinine levels were measured on an automatic biochemical analyzer (Mindray, Shenzhen, China, BS-240VET) using BUN (Mindray, 0041-30-53008) and creatinine (Mindray, 105-000456-00) assay kits.

For histology, kidney tissues were washed in cold PBS, fixed in 4% formaldehyde for 24 h, and sliced into 4-µm sections. Parts of the paraffinized sections were used for Masson’s trichrome staining (Leagene, DC0032) or SR/Fast green staining (Leagene, DC0040) following the manufacturer’s procedures. Finally, the fibrosis area (%) was measured in 10 randomly selected fields from each section using ImageJ software.

Immunohistochemistry was performed on 4-µm paraformaldehyde-fixed paraffin-embedded kidney tissue sections. Briefly, the sections were incubated in 3% hydrogen peroxide, and Tris-EDTA antigen repair solution (pH 9.0) (absin, abs9342) was used after dewaxing and rehydration. The samples were blocked with 3% bovine serum albumin blocking solution and incubated overnight at 4 °C with the following primary antibody solutions: CD11b (Abcam, ab10079; 1:250), vimentin (Proteintech, 10366-1-AP; 1:250), α-SMA (actin alpha 2, Abcam, ab5694; 1:250), or fibronectin 1 (FN1, Abcam, ab2413; 1:250). Finally, tissue staining was visualized under a microscope (Nikon, Tokyo, Japan, ECLIPSE Ci-S), and the positive area (%) was analyzed in 10 randomly selected fields from each section using ImageJ software.

### Quantitative real-time PCR

Total RNA was extracted from the kidney or isolated renal myeloid cells with TRIzol Reagent (Ambion, 15,596,026) following the manufacturer’s protocol. The RNA was reverse-transcribed into single-stranded cDNA using a commercial reverse transcription kit (Roche, 4,897,030,001). Quantitative real-time PCR was performed on the LightCycler 480 System (Roche, Basel, Switzerland) using the SYBR Green I Master kit (Roche, 4,887,352,001), following the manufacturer’s instructions. Data were calibrated to glyceraldehyde phosphate dehydrogenase values. The sequences of primers used in the experiments included *Atg5*, α-SMA (*Acta2*), Collagen 1a1 (*Col1a1*), CCR6 (*Ccr6*), CXCR2 (*Cxcr2*), CCL20 (*Ccl20*),TNFα (*Tnfα)*, Il6 (*Il-6*), iNOS (*iNOS*), TGFβ1 *(Tgfb1*), and GADPH (*Gapdh*) (Table S1).

### Isolation and treatment of BMDMs

MΦ *atg5*^*−/−*^ and WT mice were anesthetized with isoflurane, sacrificed, and soaked in 75% alcohol for 3 min for disinfection. Subsequently, the tibia and femur were harvested. Next, the tibia and femur cells were flushed using a 1-mL syringe with RPMI 1640 medium (Solarbio, 31,800) supplemented with 10% fetal bovine serum (Gibco, 10099-141). The cell suspension was passed through a 40-µm cell strainer, centrifuged, resuspended in 5 mL of erythrocyte lysis buffer (QIAGEN, 79,217), and incubated for 5 min. The cells were recentrifuged and resuspended in RPMI 1640 medium with 5 mM HEPES followed by monocyte isolation using a monocyte isolation kit for mice (Miltenyi Biotec, 130-100-629) to obtain BMDMs. Finally, the BMDMs were plated in a 6-well plate at a density of 4 × 10^6^ cells per well and stimulated with 1 µg/mL LPS or PBS for 4 h at 37 °C.

### Tracking monocyte cell migration into the injured kidney

BMDMs from MΦ *atg5*^*−/−*^ and WT mice were labeled using CellTracker™ green 5-chloromethylfluorescein diacetate (CMFDA) dye (Invitrogen, c70251). Each C57BL/6 recipient mouse was injected with 2 × 10^6^ CMFDA-labeled BMDMs from MΦ *atg5*^*−/−*^ or WT mice via the tail and subjected to unilateral renal pedicle clamping for 31 min the next day. Three days later, the animals were sacrificed, and the injured kidneys were collected for cryosections. Next, kidney macrophage infiltration was evaluated by counting double-positive labeling with CMFDA (green) and F4/80 (red) using a confocal microscope (Carl Zeiss AG, Oberkochen, Germany, LSM980), which was identified as 4’,6-diamidino-2-phenylindole^+^nuclei (DAPI, Solarbio, C0065) as described in the literature [22]. Finally, flow cytometry was used to analyze the number of macrophage migration to the injured kidney.

### Isolation of kidney macrophages

Single-cell suspensions were obtained from kidney tissues using enzymatic digestion with Liberase (Roche, 05401127001) diluted in Gey’s balanced salt solution. Fascia removal was performed using a 40-µm cell strainer. Next, F4/80^+^ cells were extracted from the cell suspension using the EasySep™ Mouse Biotin Positive Selection Kit II (STEMCELL, 17,665) according to the manufacturer’s instructions. Briefly, the kidney cells were transferred to a 5-mL (12 × 75 mm) polystyrene round-bottom tube and resuspended in RoboSep Buffer (STEMCELL, 20,104), blocked with FcR, and incubated with anti-mouse F4/80 antibody (STEMCELL, 60027BT) for 15 min. Subsequently, the cells were centrifuged, resuspended, incubated with the selection cocktail for 15 min, and reacted with RapidSpheres for 10 min. Finally, the tubes (without lids) were placed into a magnet (STEMCELL, 18,103) for 5 min, the supernatant was discarded, and the cells were collected from the sides of the tube.

### Cell migration in vitro

The in vitro migration of BMDMs from MΦ *atg5*^*−/−*^ and WT mice was evaluated using Transwells as previously described [22, 23]. Freshly isolated BMDMs (1 × 10^6^) were seeded in the top chamber of a 24-well polyester membrane (8-mm pore size; BD Biosciences, 353,097) in serum-free medium. The cells translocated to the lower chamber in response to exposure to RPMI 1640 medium containing 10% fetal bovine serum and 50 ng/mL CXCL3 (R&D Systems, 453-KC-010) or CCL20 (ORIGENE, TP723274). After incubation for 4 h, the cells in the upper chamber were gently wiped with a cotton swab, and the filter membrane was fixed with 4% methanol and stained with 0.2% crystal violet (Solarbio, g1061). Next, the filters were photographed using a microscope (OLYMPUS, Shinjuku, Tokyo, Japan, BX63), and the cell numbers were counted in each field of view under ×200 magnification.

### Western blotting

BMDMs were seeded in a 6-well plate at 3.5 × 10^6^ cells per well. Cells in the experimental group were treated with 200 ng/mL CXCL3 or CCL20 collected after 30 min incubation at 37 °C. Subsequently, the stimulated BMDMs were homogenized in RIPA lysis buffer (CWBIO, CW23335) containing protease inhibitors for 30 min at 4 °C and centrifuged at 12,000 *× g* for 15 min. The supernatant was collected as the total protein for Western blotting. The primary and secondary antibodies were as follows: β-actin (Sigma, A5361; 1:1000), AKT (Cell Signaling Technology, 4691; 1:1000), p-AKT (Cell Signaling Technology, 4060; 1:1000), ERK1/2 (Cell Signaling Technology, 9102; 1:1000), p-ERK1/2 (Cell Signaling Technology, 4307; 1:1000), PI3K (Cell Signaling Technology, 4257; 1:1000), p-PI3K (Cell Signaling Technology, 4228; 1:1000), goat anti-rabbit IgG-HRP (Asbio, As006; 1:2000), and goat anti-mouse IgG-HRP (Asbio, As005; 1:2000). ECL detection reagents (Cytiva, RPN2232) were used for color development and Tanon gel imaging, and the gray value was obtained after analysis using ImageJ software.

### Statistical analyses

All statistical analyses were performed using GraphPad Prism version 8.0.1 (GraphPad). Data are expressed as the mean ± SEM. To assess the significant differences, two-tailed unpaired Student’s *t* tests were used to compare 2 independent groups, and one-way ANOVA or two-way ANOVA was used to compare 3 or more groups. A *P* value of less than 0.05 was considered statistically significant.

## Results

### Selective ablation of *Atg5* in the myeloid cells of mice

**Fig. 1:**
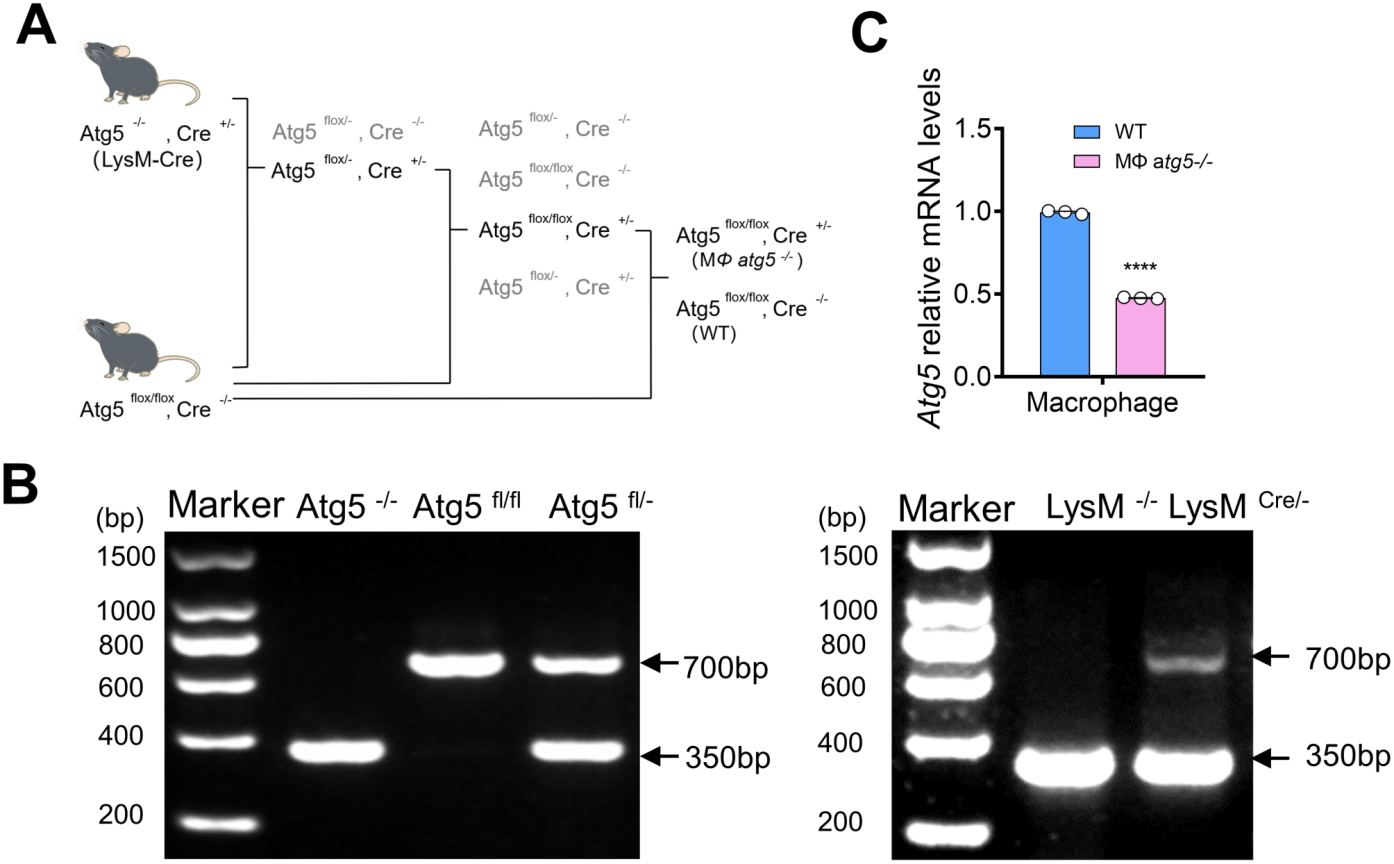
Renal myeloid autophagy-related 5 (*Atg5*) was efficiently deleted in *LysM-Cre*^*+/−*^*Atg5*^*f/f*^ (MΦ *atg5*^*−/−*^) mice. (**A**) Breeding protocol for generating *LysM-Cre*^*+/−*^*Atg5*^*f/f*^ (MΦ *atg5*^*−/−*^) and *Cre*^*−/−*^*Atg5*^*f/f*^ (WT) male littermates, aged 8–10 weeks, for subsequent experiments after genotypes were confirmed. (**B**) MΦ *atg5*^*−/−*^ mice verified by polymerase chain reaction using isolated genomic DNA. (**C**) *Atg5* mRNA levels in immunomagnetically sorted kidney macrophages from WT and MΦ *atg5*^*−/−*^ mice. Data are presented as the mean ± SEM, *n* = 3 per group, **** *P* < 0.0001 versus WT macrophages. Unpaired, two-tailed Student’s t test

*Atg5*-floxed mice were hybridized with *LysM-Cre* mice to establish conditional deletion of *Atg5.* The breeding protocol is shown in Fig. 1A. Two polymerase chain reaction (PCR) sets were conducted for each mouse to verify the genotypes. The genotype of myeloid cell-specific *Atg5* knockout (MΦ *atg5*^*−/−*^) mice was identified by amplification of the 700-bp fragment of the floxed allele, insufficient amplification of the 350-bp fragment of the wild type (WT) allele, and amplification of the 700-bp fragment of the *Cre* gene (Fig. 1B). The effectiveness of *Atg5* deletion in renal macrophages of samples isolated from MΦ *atg5*^*−/−*^ and WT mice using F4/80 + cell magnetic beads was determined using quantitative real-time PCR (Fig. 1C). These mice also showed low BUN and serum creatinine levels without surgical treatment, indicating that renal function remained normal (data not shown).

### *Atg5* deletion in myeloid cells did not affect renal function in the early stages of AKI

**Fig. 2:**
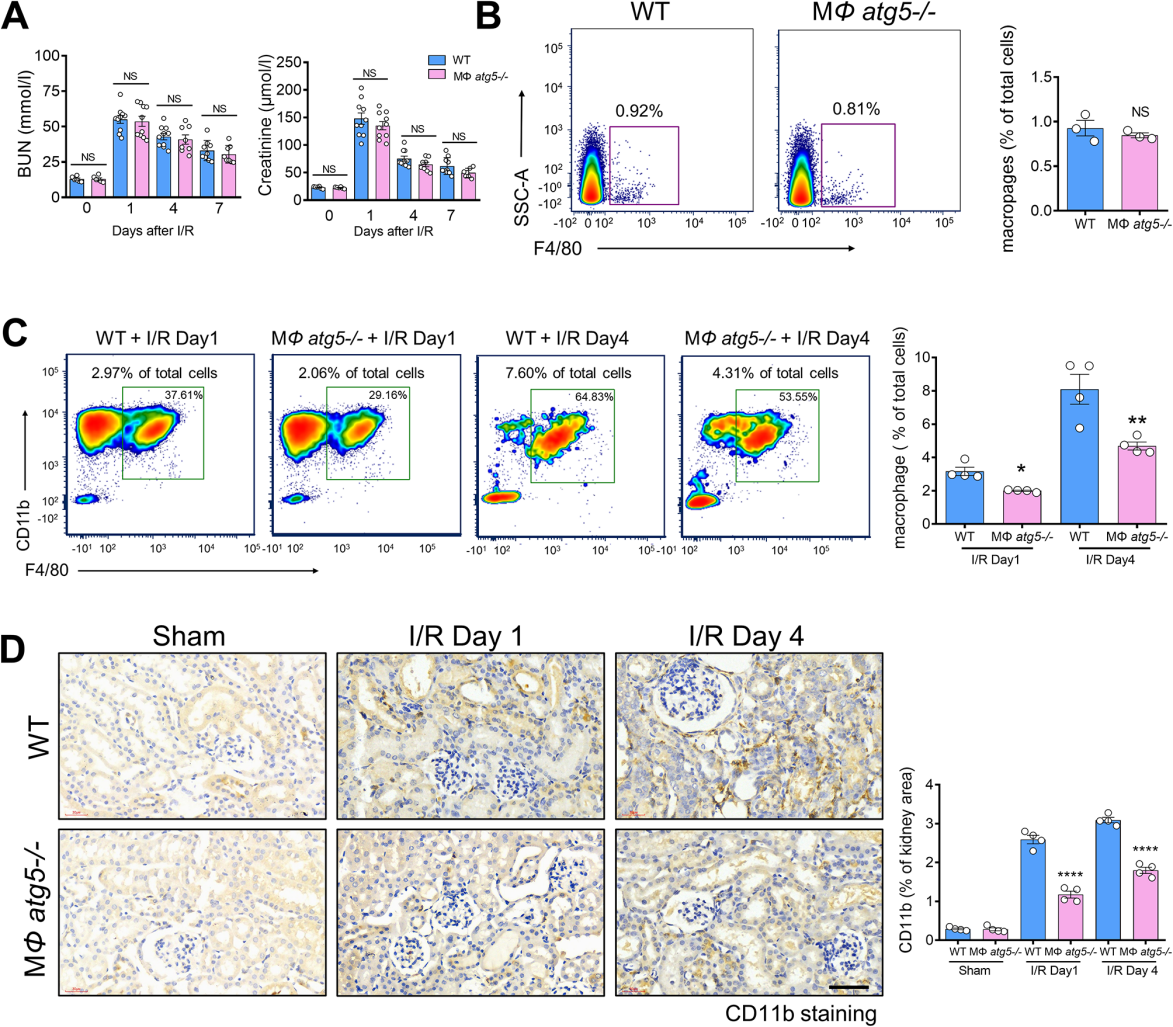
Myeloid autophagy-related 5 (*Atg5*) deletion inhibited macrophage migration into the kidneys in early acute ischemic injury. Wild-type (WT) and MΦ *atg5*^*−/−*^ mice underwent 26 min of bilateral renal ischemia/reperfusion (I/R) injury and were sacrificed at the indicated time points. (**A**) Blood urea nitrogen (BUN) and serum creatinine levels in WT and MΦ *atg5*^*−/−*^ mice at days 0, 1, 4, and 7 after I/R injury (*n* = 8–10 per group). (**B** and **C**) Flow cytometric analysis showing the number of renal macrophages (F4/80^+^ CD11b^+^ CD11c^−^ CD45^+^ cells) in MΦ *atg5*^*−/−*^ and WT mice (**B**) at baseline and (**C**) on days 1 and 4 after I/R. Data are presented as the mean ± SEM, * *P* < 0.05, ** *P* < 0.01 (*n* = 3–4 per group). (**D**) Immunohistochemical staining for CD11b on kidney sections from both experimental groups at days 1 and 4 after AKI induction. Scale bar: 50 μm. Quantitation of the levels of CD11b^+^ cells from the renal cortex of MΦ *atg5*^*−/−*^ and WT mice. Data are presented as the mean ± SEM, **** *P* < 0.0001 (*n* = 4 per group). One-way ANOVA with Tukey’s multiple-comparison test (**A**, **C**, **D**); Unpaired, two-tailed Student’s t test (**B**)

At each point during the early stage of AKI, MΦ *atg5*^*−/−*^ mice did not exhibit any changes in BUN and serum creatinine levels compared with those of the WT littermate mice (Fig. 2A), and the level of kidney inflammation and injury was visualized by the HE staining (Figure S1), indicating that *Atg5* deletion in myeloid cells did not affect renal function during early AKI. Flow cytometric analysis showed that the number of renal macrophages (F4/80^+^ CD11b^+^ CD11c^−^ cells) was similar at baseline between MΦ *atg5*^*−/−*^ and WT littermate mice; however, it was significantly decreased on days 1 and 4 after ischemic injury in KO mice (Fig. 2, B and C). Similarly, immunohistochemical staining on days 1 and 4 after AKI induction showed that the kidneys of MΦ *atg5*^*−/−*^ mice had less CD11b^+^ cells infiltration than those of WT littermate mice (Fig. 2D, Figure S2a).

### *Atg5* deletion in myeloid cells protects against renal fibrosis

**Fig. 3:**
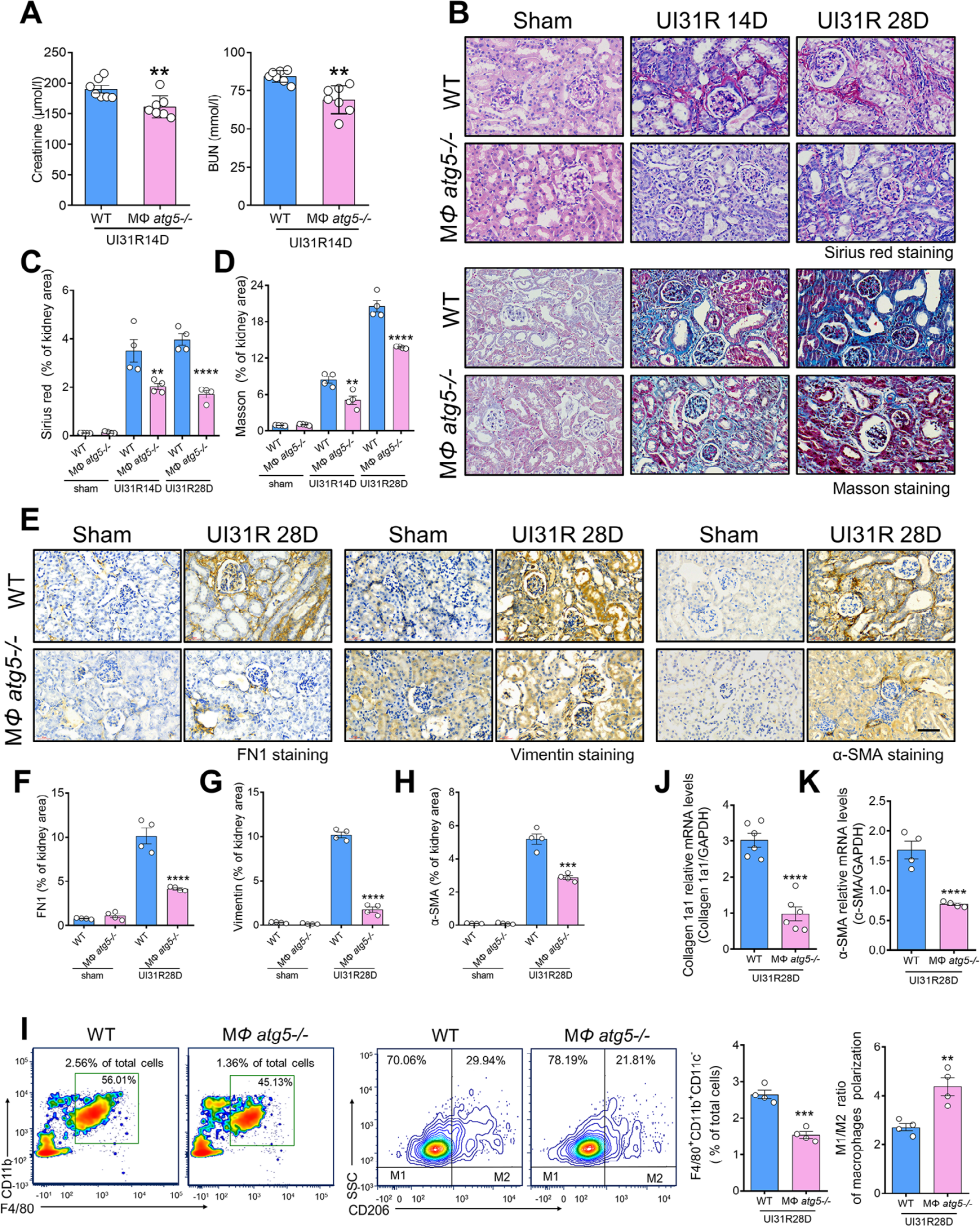
Macrophage autophagy-related 5 (*Atg5*) deletion protects against kidney fibrosis after I/R injury. (**A**) Serum creatinine and BUN levels of MΦ *atg5*^*−/−*^ and wild type (WT) mice. Mice underwent 31-min unilateral renal ischemia (UI31R) surgery, the right kidney was removed after 13 days of left renal reperfusion, and blood samples were collected 1 day later. Data are presented as the mean ± SEM. ** *P* < 0.01 (*n* = 6 per group). Unpaired, two-tailed Student’s t test. (**B-K**) WT and MΦ *atg5*^*−/−*^ mice underwent 31-min unilateral renal I/R and were sacrificed at 2 or 4 weeks. Representative images and positive area rate of Sirius red (**B** and **C**) and Masson (**B** and **D**) staining of MΦ *atg5*^*−/−*^ mouse kidney cortex sections showing reduced renal tubulointerstitial fibrosis. (**E**) Representative fibrosis immunohistochemical staining images showing decreased renal fibrosis in MΦ *atg5*^*−/−*^ mice. Renal expression of fibronectin 1 (FN1) (**F**), vimentin (**G**), and α-SMA (**H**), a marker of myofibroblasts. Scale bars: 50 μm. Data are presented as the mean ± SEM. ** *P* < 0.01, ***P < 0.001, ****P < 0.0001 (*n* = 4 per group). One-way ANOVA with Tukey’s multiple-comparison test (**C**, **D**, **F**, **G, H**). (**I**) Flow cytometric analysis showing the number of insulted renal macrophages (F4/80^+^ CD11b^+^ CD11c^−^ CD45^+^ cells) and the M1/M2 ratio of macrophages in MΦ *atg5*^*−/−*^ and WT mice on day 28 after ischemic injury. Data are presented as the mean ± SEM. ** *P* < 0.01, ***P < 0.001 (*n* = 4 per group). Unpaired, two-tailed Student’s t test. (**J** and **K**) The mRNA levels of profibrotic and fibrotic collagen 1a1 and α-smooth muscle actin (α-SMA) in insulted left kidneys of MΦ *atg5*^*−/−*^ and WT mice. Data are presented as the mean ± SEM. ****P < 0.0001 (collagen 1a1 groups, *n* = 6 each; α-SMA groups, *n* = 4 each). Unpaired, two-tailed Student’s t test

We performed unilateral renal ischemia surgery (duration, 31 min) to elucidate the specific role of *Atg5* in macrophages in the progression of renal fibrosis. After 13 days of left renal reperfusion, the right kidney was removed, and blood samples were collected 1 day later. Our test indicated that serum creatinine and albumin levels were lower in MΦ *atg5*^*−/−*^ mice than in WT littermate mice (Fig. 3A). MΦ *atg5*^*−/−*^ mice also showed reduced interstitial fibrosis as indicated using Sirius red (SR) and Masson staining after 2 or 4 weeks of 31-min unilateral renal ischemia (Fig. 3, B–D). Immunohistochemical staining for fibrotic components showed lower levels of FN1, vimentin, and α-SMA in the kidneys of MΦ *atg5*^*−/−*^ mice than in those of WT littermate mice (Fig. 3, E–H), indicating reduced severity of renal fibrosis. Additionally, we demonstrated that the counts of F4/80^+^ CD11b^+^ CD11c^−^ renal macrophages were decreased in the kidneys of MΦ *atg5*^*−/−*^ mice compared with those in WT littermate mice using flow cytometry analysis. The numbers of M1 and M2 macrophages were determined by gating on CD206^−^ F4/80^+^ CD11b^+^ CD11c^−^ cells and CD206^+^ F4/80^+^ CD11b^+^ CD11c^−^ cells, respectively. Therefore, we found that *Atg5* deletion inhibited the polarization of M1 macrophages toward M2 macrophages at 28 days after ischemic injury (Fig. 3I). Additionally, quantitative real-time PCR revealed lower renal mRNA levels of collagen 1a1 and α-SMA at 4 weeks after I/R in MΦ *atg5*^*−/−*^ mice than in WT littermate mice (Fig. 3, J and K, Figure S2b, c).

**Fig. 4:**
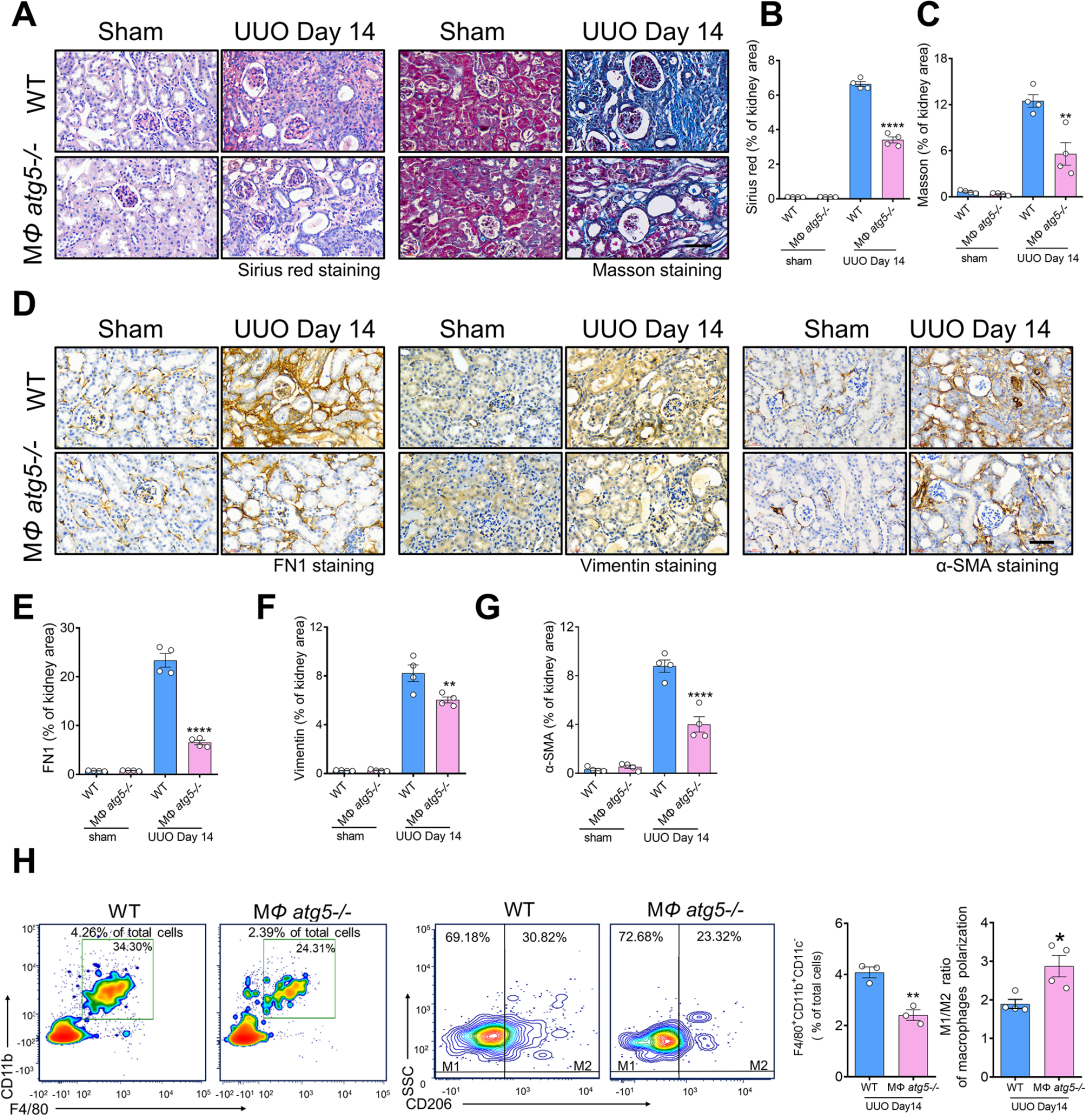
Macrophage autophagy-related 5 (*Atg5*) deletion protected against renal fibrosis in a unilateral ureteral obstruction (UUO) mouse model. (**A-H**) UUO was induced in WT and MΦ *atg5*^*−/−*^ mice for 2 weeks. Representative images and positive area rate of Sirius red (**A** and **B**), Masson (**A** and **C**), and immunohistochemical staining for FN1 (**D** and **E**), vimentin (**D** and **F**), and α-SMA (**D** and **G**) showing less renal fibrosis in MΦ *atg5*^*−/−*^ mice than in WT mice. Data are presented as the mean ± SEM. * *P* < 0.05, ** *P* < 0.01, ****P < 0.0001 (*n* = 4 per group). One-way ANOVA with Tukey’s multiple-comparison test. (**H**) Flow cytometric analysis showing renal macrophages (F4/80^+^ CD11b^+^ CD11c^−^ CD45^+^ cells) and the M1/M2 ratio of macrophages in the obstructed kidneys from MΦ *atg5-/-* mice and WT mice. Data are presented as the mean ± SEM. * *P* < 0.05, ** *P* < 0.01 (*n* = 4 per group). Unpaired, two-tailed Student’s t test

Similarly, 14 days after UUO, the kidneys of MΦ *atg5*^*−/−*^ mice revealed less renal fibrosis, as indicated using quantitative SR and Masson staining (Fig. 4, A–C), and reduced staining for fibrotic components (FN1, vimentin, and α-SMA) compared to WT littermate mice (Fig. 4, D–G). We also found lower numbers of F4/80^+^ CD11b^+^ CD11c^−^ macrophages and a higher ratio of M1/M2 macrophages in the obstructed kidneys of MΦ *atg5*^*−/−*^ mice than in the kidneys of WT littermate mice (Fig. 4H, Figure S2d).

Therefore, the above results from the two independent mouse models of CKD indicate that MΦ *atg5*^*−/−*^ has protective effects against kidney fibrosis.

### Myeloid *Atg5* deletion decreased macrophage migration in vivo

**Fig. 5:**
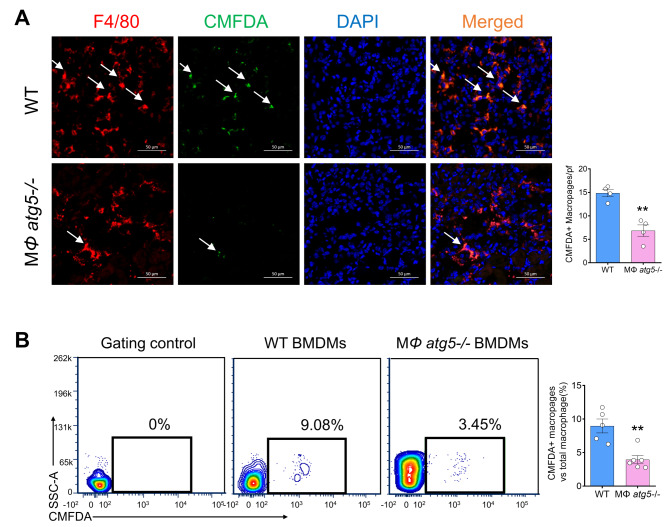
Macrophage autophagy-related 5 (*Atg5*) deletion attenuated macrophage renal infiltration in vivo. Bone marrow-derived monocytes (BMDMs) were isolated from WT and MΦ *atg5*^*-/-*^ mice, labeled with the tracking dye 5-chloromethylfluorescein diacetate (CMFDA), and injected into WT mice; unilateral acute kidney injury was induced the next day. The labeled cells in the kidney were evaluated 3 days after ischemic injury. (**A**) Representative image of injured kidneys and the number of CMFDA and F4/80 double-positive renal-infiltrated cells in mice receiving *Atg5* knockout (KO) and WT monocytes. Scale bar: 50 μm. Data are presented as the mean ± SEM. ** *P* < 0.01 (*n* = 4 per group). (**B**) Flow cytometric analysis to determine the F4/80 and CMFDA double-positive cell numbers in mice receiving *Atg5* KO and WT monocytes. Data are presented as the mean ± SEM. ** *P* < 0.01 (*n* = 6 per group). Unpaired, two-tailed Student’s t test

To investigate the potential role of macrophage *Atg5* expression in macrophage migration to the injured kidney, we obtained BMDMs from WT or MΦ *atg5*^*−/−*^ mice, labeled them using green CMFDA dye, injected 2 × 10^6^ CMFDA-labeled BMDMs into WT littermate mice with induced unilateral I/R injury 16 h prior, and evaluated the number of CMFDA-labeled F4/80 double-positive cells in the kidney 3 days after AKI induction. As indicated in Fig. 5A, significantly fewer double-labeled macrophage *atg5*^*−/−*^ cells than double-labeled WT cells were found in the injured kidneys. Flow cytometry also showed that the number of double-positive cells in the kidneys of mice injected with CMFDA probe-labeled *atg5* KO BMDMs decreased compared with that of mice injected with WT BMDMs (*P* < 0.01) (Fig. 5B).

### *Atg5* deficiency in macrophages impairs the CCL20-CCR6 axis in experimental kidney fibrosis models

**Fig. 6:**
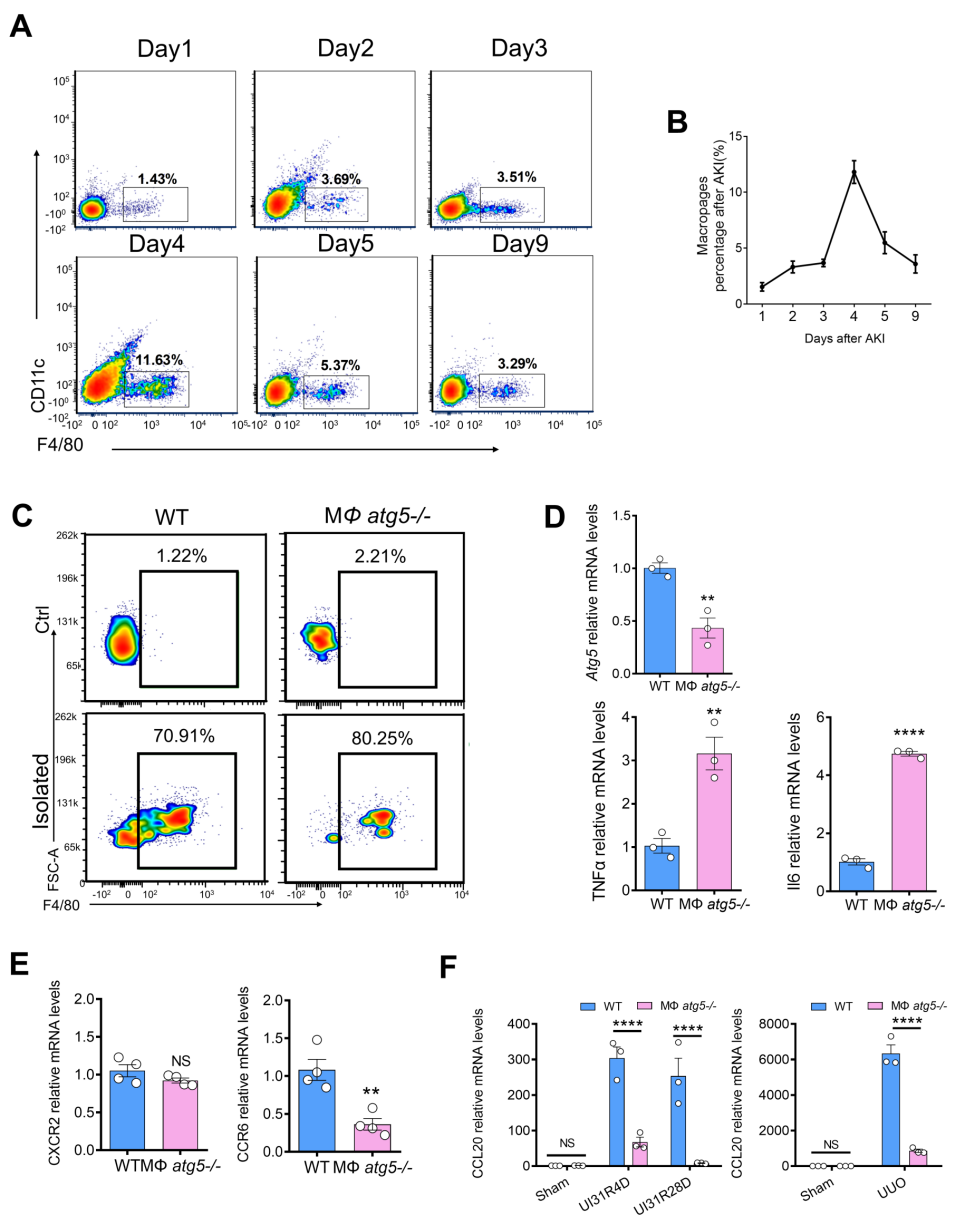
Macrophage autophagy-related 5 (*Atg5*) deletion impaired the CCR6-CCL20 axis after I/R and UUO injury. (**A** and **B**) Flow cytometric analysis determined that F4/80^+^ macrophage infiltration injured kidneys at 1, 2, 3, 4, 5, and 9 days after undergoing 31-min unilateral renal ischemia/reperfusion (UI31R). (**C**, **D** and **E**) MΦ *atg5*^*−/−*^ and WT mice were sacrificed 4 days after UI31R injury. (**C**) F4/80^+^ macrophages isolated from kidney single-cell suspensions from MΦ *atg5*^*−/−*^ and WT mice using immunomagnetic positive selection. Flow cytometric analysis identified the isolated cell purity. (**D** and **E**) The renal macrophage mRNA levels of *Atg5*, TNFα, Il6, CCR6 and CXCR2 in MΦ *atg5*^*−/−*^ and WT mice. Data are presented as the mean ± SEM. ** *P* < 0.005, **** *P* < 0.0001 (*n* = 3–4 per group). Unpaired, two-tailed Student’s t test. (**F**) The renal mRNA level of CCL20 was significantly lower in MΦ *atg5*^*−/−*^ mice than in WT mice at 4 and 28 days after UI31R injury and 14 days after UUO injury. Data are presented as the mean ± SEM. **** *P* < 0.0001 (*n* = 3 per group). Two-way ANOVA with Sidak’s multiple-comparison test

In this study, we explored the mechanism by which *Atg5* mediates macrophage migration to the postischemic kidney. Chemokines are known to mediate the infiltration of immune cells into injured tissue, and many studies have shown that autophagy is associated with the CCL20-CCR6 axis. Therefore, we hypothesized that *Atg5* deficiency inhibits macrophage migration by impairing the CCL20-CCR6 axis. Infiltration of F4/80^+^ cells (macrophages) in the kidneys of healthy male C57BL/6 mice peaked 4 days after I/R injury (Fig. 6, A and B). We isolated infiltrating F4/80^+^ cells (macrophages) from the kidneys of MΦ *atg5*^*−/−*^ mice after 4 days of reperfusion following unilateral renal pedicle clamping-induced ischemia for 31 min. Flow cytometric analysis showed that the isolated cell purity was ˃70% (Fig. 6C). Quantitative real-time PCR revealed that atg5 deficient reduced mRNA levels of *Atg5* and CCR6, while increased mRNA levels of TNFα and Il6 in isolated renal macrophages (Fig. 6, D-E, Figure S2c). CCR6 is considered the only receptor for CCL20. Increased CCL20 drives the recruitment of CCR6^+^ immune cells into damaged tissues. Next, we confirmed that the renal expression of CCL20 was significantly decreased in MΦ *atg5*^*−/−*^ mice compared with WT mice at 4 and 28 days after AKI and 14 days after UUO (Fig. 6F, Figure S2d). Therefore, these results indicate that *Atg5*-deficient macrophages impaired the CCL20-CCR6 axis in experimental kidney fibrosis models.

### Myeloid *Atg5* deletion decreased the migration response to CCL20 chemokines

**Fig. 7:**
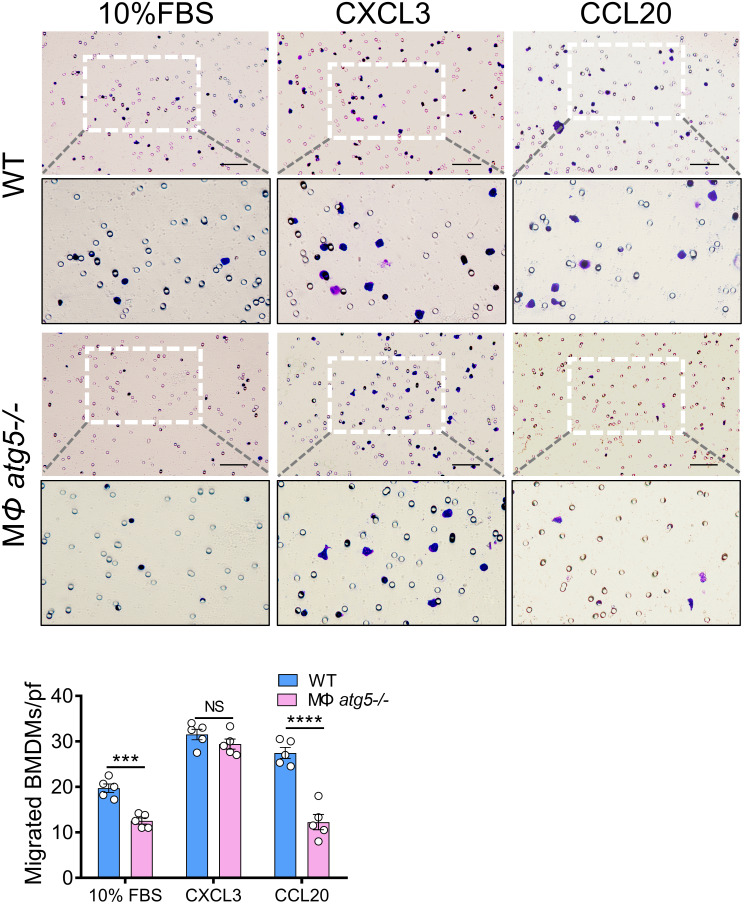
Macrophage autophagy-related 5 (*Atg5*) deletion decreased chemotactic responses to CCL20 in BMDMs in vitro. BMDMs were isolated from MΦ *atg5*^*−/−*^ mice and WT mice and immediately used for in vitro Transwell migration assays. Chemotactic responses to 10% FBS, CXCL3, and CCL20 in MΦ *atg5*^*−/−*^ and WT BMDMs. Data are presented as the mean ± SEM. *** *P* < 0.001, **** *P* < 0.0001 for WT BMDMs versus MΦ *atg5*^*−/−*^ BMDMs (*n* = 5 per group). Two-way ANOVA with Sidak’s multiple-comparison test

To investigate the impact of *Atg5* deletion on the CCL20 chemokine response of BMDMs, we selected chemokine (C-X-C motif) ligand 3 (CXCL3) [ligand of CXC chemokine receptor 2 (CXCR2)] as a control [23] based on the data that CXCR2 expression from the isolated infiltration of F4/80^+^ cells (macrophages) in the kidneys was not different between the MΦ *atg5*^*−/−*^ and WT mice 4 days after severe AKI (Fig. 6E). The chemotactic response to CCL20 was significantly inhibited in BMDMs from MΦ *atg5*^*−/−*^ mice compared with BMDMs from WT mice. However, the response to CXCL3 was not different in BMDMs from MΦ *atg5*^*−/−*^ mice compared with that of BMDMs from WT mice. Our results indicated that *Atg5* deficiency markedly decreased the chemotactic response to CCL20 in BMDMs from MΦ *atg5*^*−/−*^ mice due to the low expression of CCR6, suggesting that the inhibition of the CCL20-CCR6 axis decreased the migration of *Atg5-*deficient macrophages (Fig. 7).

### *Atg5* deletion inhibits the PI3K/AKT or ERK pathway from affecting macrophage migration

**Fig. 8:**
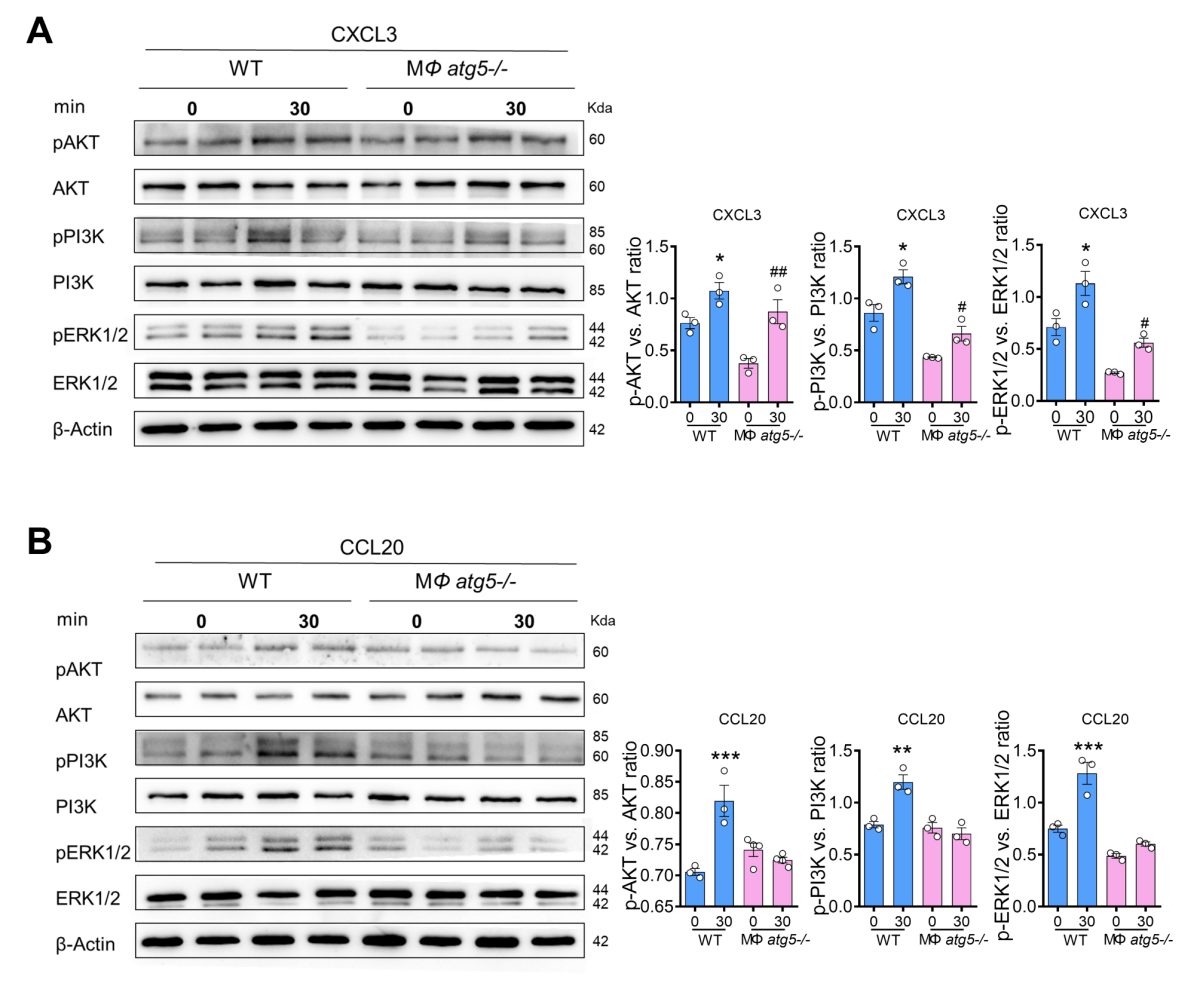
Macrophage autophagy-related 5 (*Atg5*) deletion inhibited the PI3K-AKT pathway. BMDMs from MΦ *atg5*^*−/−*^ and WT mice were treated with 200 ng/mL CXCL3 or CCL20 for 0–30 min. Western blot analysis of the activation of the AKT, PI3K, and ERK pathways in WT and MΦ *atg5*^*−/−*^ BMDMs following (**A**) CXCL3 and (**B**) CCL20 treatment. Data are presented as the mean ± SEM. * *P* < 0.05, ** *P* < 0.01, *** *P* < 0.001 for 0 versus 30 min treatment in WT BMDMs, #*P* < 0.05, ## *P* < 0.01 for 0 versus 30 min treatment in MΦ *atg5*^*−/−*^ BMDMs (*n* = 3 per group). Two-way ANOVA with Sidak’s multiple-comparison test

It is known that activation of phosphoinositide-3-kinase-protein kinase B (PI3K-AKT) or mitogen-activated protein kinases-extracellular signal-regulated kinase (MAPK/ERK) is involved in cell migration [24]. The treatment of CXCL3 resulted in the activation of PI3K, AKT, and ERK (increased phosphorylation levels) in WT and MΦ *atg5*^*−/−*^ BMDMs. However, CCL20 treatment markedly attenuated the activation of AKT, PI3K, and ERK in MΦ *atg5*^*−/−*^ BMDMs compared to WT BMDMs. These results suggest that the reduced CCL20 chemokine responses in MΦ *atg5*^*−/−*^ BMDMs inhibited PI3K, AKT, and ERK phosphorylation (Fig. 8, A and B).

### Inflammatory cytokines characteristic of MΦ *atg5*^*−/−*^ BMDMs respond to LPS

**Fig. 9:**
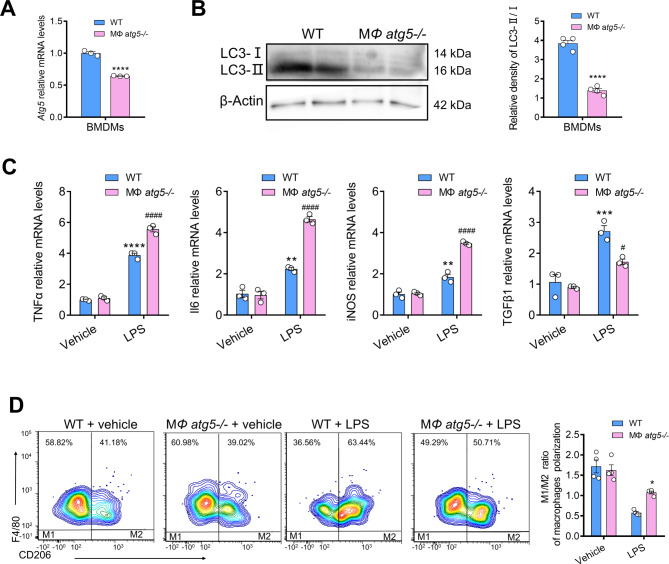
Macrophage autophagy-related 5 (*Atg5*) deletion alters the inflammatory phenotype and polarization in BMDMs in response to lipopolysaccharides (LPS). (**A**) *Atg5* mRNA levels in BMDMs from WT and MΦ *atg5*^*−/−*^ mice. Data are presented as the mean ± SEM, *n* = 3 per group, **** *P* < 0.0001. Unpaired, two-tailed Student’s t test. (**B**) Western blot analysis of the relative density of LC3-II;/I; in WT and MΦ *atg5*^*−/−*^ BMDMs following stimulation with 1 µg/mL LPS for 4 h. Data are presented as the mean ± SEM. Data are presented as the mean ± SEM, *n* = 4 per group, **** *P* < 0.0001. Unpaired, two-tailed Student’s t test. (**C**) The mRNA levels of TNFα, Il6, iNOS, and TGFβ1 in MΦ *atg5*^*−/−*^BMDMs compared with those of WT BMDMs following stimulation with 1 µg/mL LPS. Data are presented as the mean ± SEM. ** *P* < 0.01, *** *P* < 0.001 **** *P* < 0.0001 versus vehicle-treated WT BMDMs; *## P* < 0.01, *#### P* < 0.0001 for LPS-treated WT BMDMs (*n* = 4 per group). (**D**) Flow cytometric analysis showing the M1/M2 ratio of macrophage polarization in BMDMs isolated from MΦ *atg5*^*−/−*^ and WT mice in response to LPS treatment. Data are presented as the mean ± SEM. *P < 0.05 (*n* = 4 per group). Two-way ANOVA with Sidak’s multiple-comparison test

We examined whether MΦ *atg5*^*−/−*^ BMDMs (isolated from MΦ *atg5*^*−/−*^ mice) alter inflammation in response to 4 h of LPS stimulation. We first confirmed that autophagy levels (Atg5 and LC3II/I;) were significantly reduced in MΦ *atg5*^*−/−*^ BMDMs (Fig. 9, A and B), and then quantitative real-time PCR revealed that gene expression of proinflammatory cytokines, such as TNFα, Il6, and iNOS, was increased in LPS-treated MΦ *atg5*^*−/−*^ BMDMs at a dose of 1 µg/mL compared to WT BMDMs (*P* < 0.0001). However, the gene expression of profibrotic cytokines such as TGFβ1 was inhibited in MΦ *atg5*^*−/−*^ BMDMs after stimulation with 1 µg/mL LPS (*P* < 0.05). The results showed that *Atg5* deficiency promoted the secretion of proinflammatory cytokines but inhibited the secretion of profibrotic cytokines. We also confirmed that the M1/M2 ratio of macrophage polarization (CD206^+^ F4/80^+^ CD11b^+^ CD11c^−^:CD206^−^ F4/80^+^CD11b^+^ CD11c^−^) was increased after LPS treatment in MΦ *atg5*^*−/−*^ BMDMs, as assessed using flow cytometry (Fig. 9, C-D).

## Discussion

This study showed that *Atg5* deletion in myeloid cells did not affect renal function in the early stages following mild ischemic injury but inhibited renal fibrosis development after severe AKI. Myeloid-specific *Atg5* deletion also reduced macrophage recruitment to the injured kidneys in vivo at each time point after AKI and inhibited the macrophage migration capacity in vitro. However, treatment with CCL20 significantly inhibited the chemotactic response of BMDMs from MΦ *atg5*^*−/−*^ mice compared with those from WT mice, which was associated with PI3K-AKT signaling pathway activation. Moreover, we stimulated the BMDMs of MΦ *atg5*^*−/−*^ mice and WT mice with LPS and found that *Atg5* deletion inhibited LPS-induced profibrotic cytokine (TGFβ1) expression but increased proinflammatory cytokines (Il6,TNFα, and iNOS), indicating that *Atg5* deletion promoted macrophages into an antifibrotic phenotype.

Our results showed that renal macrophage migration was significantly lower throughout the experiment in MΦ *atg5*^*−/−*^ mice than in WT mice. Macrophages are well known to be recruited by chemokines and accumulate to participate in renal pathogenesis [25, 26]. The CCR6-CCL20 axis participates in the chemoattraction of immune cells, and increased CCL20 drives the recruitment of CCR6^+^ immune cells into the injured kidney and plays an important role in kidney injury and repair in AKI and CKD. During AKI, kidney CCL20 is increased in damaged tubules and interstitial cells, and some studies have reported a relationship between tubular and urinary CCL20 and the severity of AKI in humans [27, 28]. Consistently, we also observed CCL20 upregulation in the kidneys of AKI and UUO mice. Therefore, we demonstrated for the first time that *Atg5* deficiency decreased CCR6 expression in macrophages to postischemic kidneys. More importantly, we found that renal CCL20 mRNA level was significantly lower in MΦ *atg5*^*−/−*^ mice than in WT mice at 28 days after AKI, which was also observed 14 days after UUO. However, lower CCL20 levels may further weaken macrophage recruitment. In vitro migration showed that the CCL20 chemoattractant was inhibited in *atg5*^*−/−*^ BMDMs, whereas the CXCL3 chemoattractant was not different between MΦ *atg5*^*−/−*^ and WT mice, suggesting that the CCR6-CCL20 axis was required for macrophage migration to the site of the injured kidney (Figure S3).

Autophagy is a dynamic cellular degradation process that maintains intracellular homeostasis by eliminating dysfunctional proteins and organelles using autolysosomes [14]. A considerable amount of evidence has shown that autophagy-dependent motility or migration varies with context-specific or different disease stages [29–33]. In solid tumor studies, the knockdown of *Atg5* and *Atg7* attenuated the recruitment of monocytes/macrophages [34]. However, the suppression or promotion of macrophage migration into the injured kidney by autophagy is still unclear. Evidence has shown that autophagy-related genes are associated with the CCL20-CCR6 axis [35–37]. In this experiment, we found that macrophage migration was decreased in myeloid *Atg5*-deleted mice, which was associated with impairment of the CCL20-CCR6 axis. Additionally, some studies found that diminished chemokine responses impair the PI3K-AKT signaling pathway and inhibit macrophage recruitment [24, 38].Consistently, our results confirmed that reduced CCL20 chemokine responses in MΦ *atg5*^*−/−*^ BMDMs inhibited PI3K, AKT, and ERK phosphorylation.

Autophagy can regulate M1/M2 macrophage polarization. However, in acute liver injury, macrophage autophagy inhibits the number of M1 macrophages and promotes M2 polarization [39, 40]. In obese mice, macrophage autophagy was generally impaired, and this increased pro-inflammatory M1 levels and decreased anti-inflammatory M2 polarization [41]. However, isoproterenol promoted the polarization of M2 macrophages by downregulating autophagy [42], and *Brucella* infection was found to inhibit the polarization of M1 and M2 macrophages by inducing autophagy associated with microtubule-associated protein 1 light chain 3 beta [43]. In a pulmonary fibrosis model, blocking macrophage autophagy protected against alveolar fibrosis by regulating the polarization of macrophages toward the M2 phenotype [44]. Zhang et al. found that the activation of macrophage autophagy by rapamycin can inhibit M1 macrophage polarization and increase renal fibrosis progression [45]. Therefore, the discrepancy in the above results is possibly due to the following reasons: different disease types, tissue specialties, time phases, and insufficient specific and accurate autophagy interventions. In our in vivo study, although flow cytometry results revealed that the M1/M2 ratio of macrophages was increased by *Atg5* deficiency, the absolute numbers of both M1 and M2 macrophages were decreased.

Additionally, BMDMs from MΦ *atg5*^*−/−*^ mice promoted M1 polarization of macrophages in response to LPS stimulation in our in vitro study: *Atg5* deletion increased LPS-induced proinflammatory factors (Il6, TNF α, and iNOS) and inhibited profibrotic cytokine (TGFβ1) expression. Previous studies have shown that macrophage-derived TGFβ1 promotes renal fibrosis [46–48]. Therefore, our study confirmed that *Atg5* deficiency promoted the conversion of macrophages to an antifibrotic phenotype, consequently alleviating renal fibrosis. It is well established that the classification of M2 macrophages is complex. However, based on the secretion of different cytokines, M2 macrophages can be subdivided into M2a, M2b, M2c, and M2d [49]. Although some studies suggest that the CD206^+^ subset of M2 macrophages is related to renal fibrosis, scarce information exists regarding the activated marker of M2, which is a particular phenotype that exacerbates or improves fibrosis [25, 50]. Notably, many intermediate phenotypes and subgroups may coexist in renal tissue. However, current technology still does not allow precise differentiation between these diverse functional subgroups of CKD progression.

## Conclusions

In summary, this study revealed that specific deletion of *Atg5* in myeloid cells protects against renal fibrosis development by attenuating macrophage migration into the injured kidney. Decreased recruitment of *Atg5* KO macrophages is largely due to the impaired CCL20-CCR6 axis, consequently inhibiting the PI3K-AKT pathway. However, the detailed mechanism by which *Atg5*-mediated macrophage migration depends on the CCL20-CCR6 axis requires further investigation. Nevertheless, these findings demonstrate that *Atg5* deletion reduces macrophage migration and changes macrophages into an antifibrotic phenotype that improves fibrosis. Therefore, our findings should be further verified in humans in clinical settings. Furthermore, we predict that there will be different responses between humans and rodents by virtue of the differences in immune cell proportions and markers. However, exploring interventions for specific autophagy and related pathways will hopefully further clarify the complex role of autophagy in immune cells and promote the application of these findings in treating AKI and CKD in the future.

### Electronic supplementary material

Below is the link to the electronic supplementary material.

Supplementary Material 1

## Data Availability

No datasets were generated or analysed during the current study.

## Abbreviations

AKI: acute kidney injury
AKT: thymoma viral proto-oncogene 1
*Atg5*: autophagy related 5
BMDMs: bone marrow-derived monocytes
BUN: blood urea nitrogen
CCL20: Chemokine (C-C motif) ligand 20
CKD: chronic kidney disease
CMFDA: 5-chloromethylfluorescein diacetate
CXCR: chemokine (C-X-C motif) receptor
ERK: extracellular signalregulated protein kinase
FN1: fibronectin 1
I/R: ischemia-reperfusion
Il: interleukin
iNOS: inducible nitric oxide synthase
KO: knockout
LPS: lipopolysaccharide
MAPK: mitogen-activated protein kinase
*MΦ atg5*^−/−^: myeloid cell-specific *atg5* knockout
PBS: phosphate-buffered saline
PCR: polymerase chain reaction
PI3K: phosphoinositide-3-kinase regulatory subunit 1
SR: Sirius red; TGFβ1:transforming growth factor β1
TNFα: tumor necrosis factor
UUO: unilateral ureteral obstruction
WT: wild-type
α-SMA: actin alpha 2
