# Supplementary material for: Atg5 deficiency in macrophages protects against kidney fibrosis via the CCR6-CCL20 axis
Source: Cell Commun Signal. 2024 Apr 9;22:223. doi: 10.1186/s12964-024-01600-2 (PMC11003172; doi:10.1186/s12964-024-01600-2)
Supplement: Supplementary file 1 — Supplementary Material 1 [file 12964_2024_1600_MOESM1_ESM.docx]

**Supplementary material**

**Supplementary Figure.**

**Supplement Figure S1.** **The HE staining of kidney sections.**

**
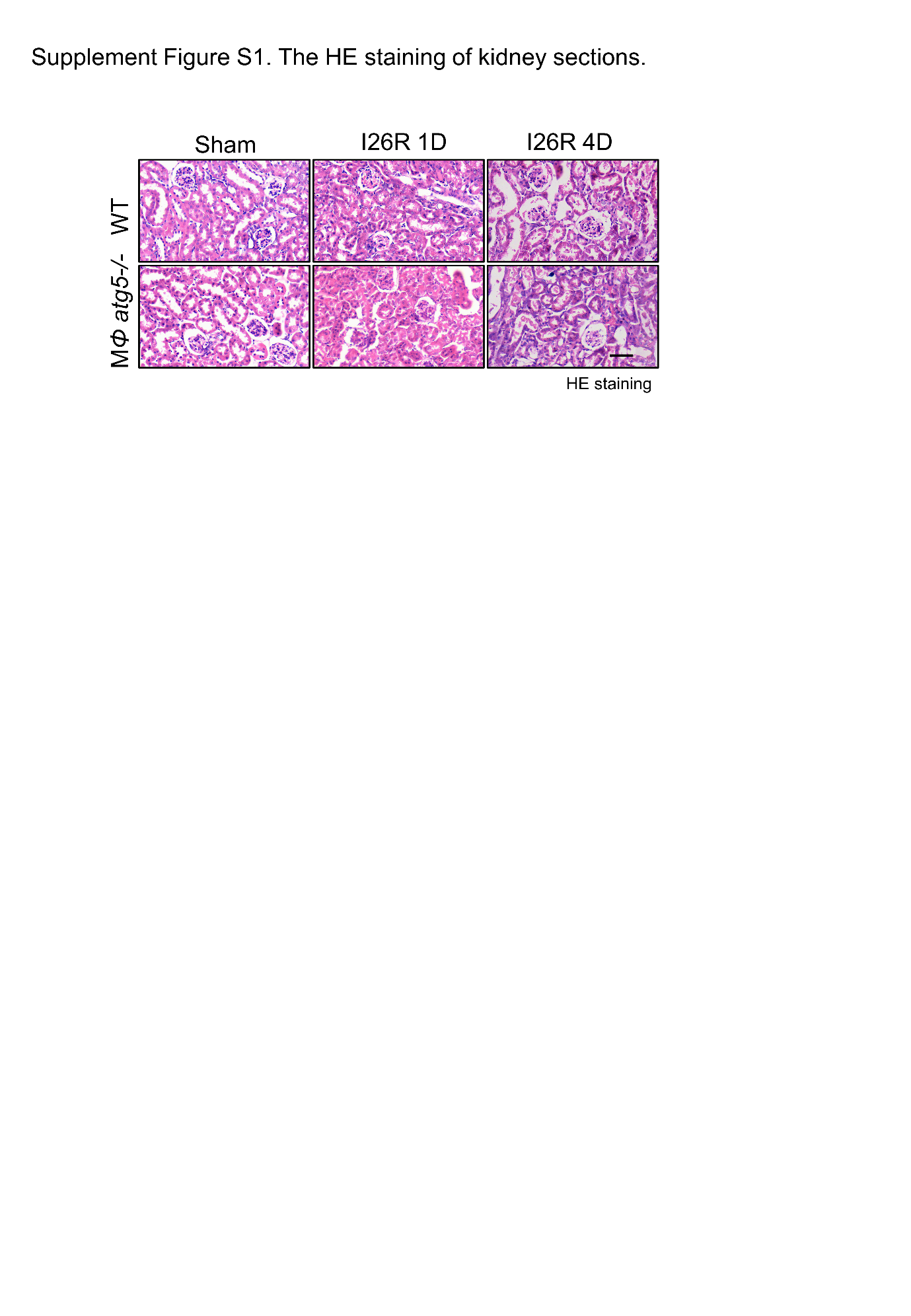
**

**Supplement Figure S1. The early renal injury caused by I/R induction.** The HE staining of kidney sections from Wild-type (WT) and MΦ *atg5^-/-^* mice underwent 26 min of bilateral renal ischemia/reperfusion (I/R) injury and were sacrificed at days 1 and days 4**.** Scale bar: 50 μm.

**Supplement Figure S2. The flowchart of the experiment.**

**
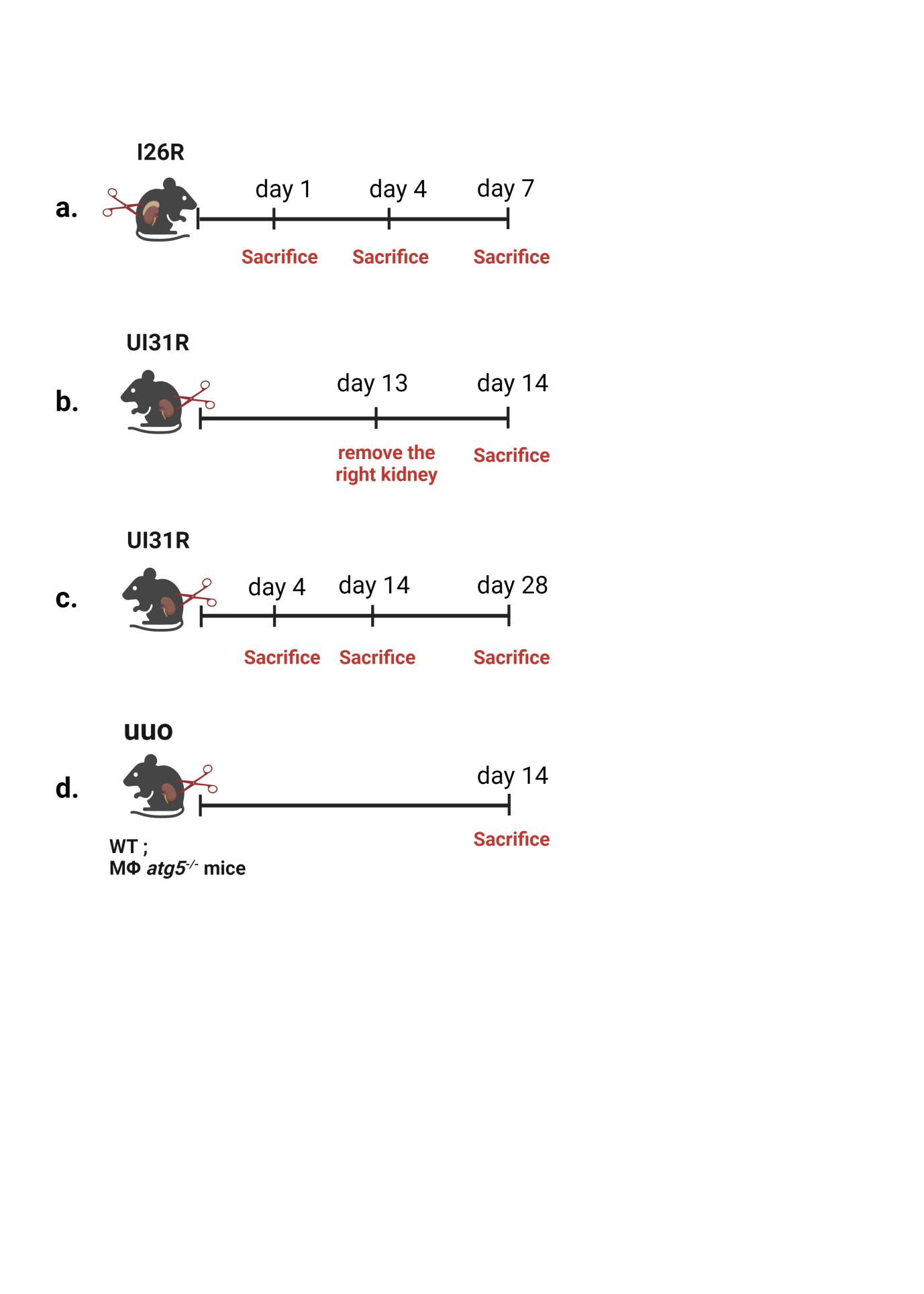
**

**Supplement Figure S2. The flowchart of of experiments for establishment of I/R and UUO mouse models. a.** Wild-type (WT) and MΦ *atg5^-/-^* mice underwent 26 min of bilateral renal ischemia/reperfusion (I26R) injury and were sacrificed at 1, 4 or 7 days. **b.** WT and MΦ *atg5^-/-^* mice underwent 31-min unilateral renal ischemia (UI31R) surgery, the right kidney was removed after 13 days of left renal reperfusion, and the mice sacrificed 1 day later. **c.** WT and MΦ *atg5^-/-^* mice underwent 31-min unilateral renal I/R and were sacrificed at 4, 14 or 28 days. **d.** Unilateral ureteral obstruction (UUO) was induced in WT and MΦ *atg5^-/-^* mice for 14 days. The flowchart was created with BioRender.com.

**Supplement Figure S3. The Schematic diagram.**

**
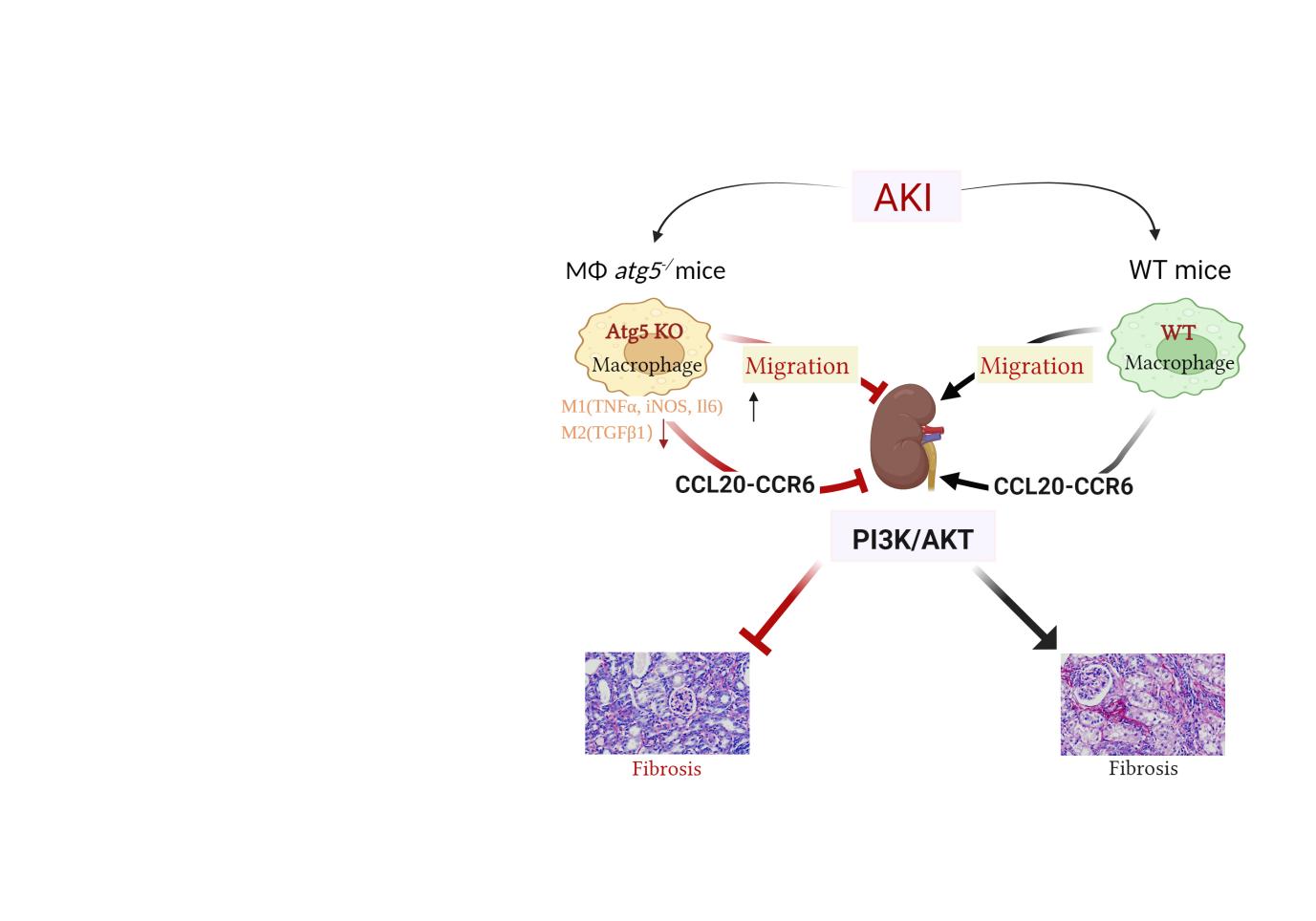
**

**Supplement Figure S3. Schematic diagram of how Atg5 deficient macrophages improve renal fibrosis.** Atg5 deficiency reduces the migration of macrophages to damaged kidneys through the CCL20-CCR6 axis and is associated with activation of the PI3K-AKT signaling pathway. In addition, Atg5 deficiency inhibited M2 polarization. These mechanisms ultimately alleviate renal fibrosis after severe acute kidney injury. The Schematic diagram was created with BioRender.com.

**Supplementary Table S1**

**Table S1. Primer sequences.**

| Gene Name | Primer sequence (5' to 3') | |
| --- | --- | --- |
| *Atg5* | F: GAAGGCACACCCCTGAAATG | R: GCTGAACTTGATGCAAGAAGATC |
| *Acta2* | F: ACTGGGACGACATGGAAAAG | R: GTTC AGTGGTGCCTCTGTCA |
| *col1a1* | F: ACATGTTCAGCTTTGTGGACC | R: TAGGCCATTGTGTATGCAGC |
| *Ccr6* | F: CCTGGGCAACATTATGGTGGT | R: CAGAACGGTAGGGTGAGGACA |
| *Cxcr2* | F: ATGCCCTCTATTCTGCCAGAT | R: GTGCTCCGGTTGTATAAGATGAC |
| *Ccl20* | F: GCCTCTCGTACATACAGACGC | R: CCAGTTCTGCTTTGGATCAGC |
| *Tnfα* | F: GCTGAGCTCAAACCCTGGTA | R: CGGACTCCGCAAAGTCTAAG |
| *Il6* | F: CACAAGTCCGGAGAGGAGAC | R: TTGCCATTGCACAACTCTTT |
| *iNos* | F: CCAAGCCCTCACCTACTTCC | R: CTCTGAGGGCTGACACAAGG |
| *Tgfβ1* | F: CAATTCCTGGCGATACCTCAG | R: GCACAACTCCGGTGACATCAA |
| *Gapdh* | F: GCCATCACTGCCACCCAGAA | R: GCCAGTGAGCTTCCCGTTGA |
